# Pneumococcal Vaccination for Children in Asian Countries: A Systematic Review of Economic Evaluation Studies

**DOI:** 10.3390/vaccines8030426

**Published:** 2020-07-30

**Authors:** Neily Zakiyah, Widya N. Insani, Auliya A. Suwantika, Jurjen van der Schans, Maarten J. Postma

**Affiliations:** 1 Department of Pharmacology and Clinical Pharmacy, Faculty of Pharmacy, Padjadjaran University, Bandung 40132, Indonesia; widya.insani@unpad.ac.id (W.N.I.); auliya@unpad.ac.id (A.A.S.); 2 Center of Excellence in Higher Education for Pharmaceutical Care Innovation, Padjadjaran University, Bandung 40132, Indonesia; m.j.postma@rug.nl; 3 Research Department of Practice and Policy, School of Pharmacy, University College London, London WC1N 1AX, UK; 4 Center for Health Technology Assessment, Universitas Padjadjaran, Bandung 40132, Indonesia; 5 Unit of Global Health, Department of Health Sciences, University Medical Center Groningen, University of Groningen, 9713 AV Groningen, The Netherlands; j.van.der.schans@rug.nl; 6 Unit of PharmacoTherapy, Epidemiology and Economics (PTE2), Department of Pharmacy, University of Groningen, 9713 AV Groningen, The Netherlands; 7 Department of Economics, Econometrics and Finance, Faculty of Economics and Business, University of Groningen, 9747 AE Groningen, The Netherlands

**Keywords:** Asia, cost analysis, cost-effectiveness, children, pneumococcal conjugate vaccine

## Abstract

*Background:* Evidence on costs and health benefits of pneumococcal conjugate vaccine (PCV) for children in Asian countries is limited but growing. As a region with a considerably high burden of pneumococcal disease, it is prominent to have a comprehensive overview on the cost-effectiveness of implementing and adopting a PCV vaccination program. *Methods:* We conducted a systematic review from Pubmed and Embase to identify economic evaluation studies of PCV for children in Asian countries up to May 2020. Data extraction included specific characteristics of the study, input parameters, cost elements, cost-effectiveness results, and key drivers of uncertainty. The Preferred Reporting Items for Systematic Reviews and Meta Analyses (PRISMA) statement was followed for this systematic review. The reporting quality of the included studies was evaluated using the Consolidated Health Economic Evaluation Reporting Standards (CHEERS) statement. *Results:* After the screening process on both the title and abstract and full text of 518 records, a total of 25 studies fulfilled the inclusion criteria, and were included in the review. The majority of included studies demonstrates that PCV for children is cost-effective in most of the Asian region, and even cost-saving in some countries. Most of the included studies implemented cost utility analysis (CUA) using either quality-adjusted life years (QALYs) or disability-adjusted life years (DALYs). Overall, the main drivers affecting the cost effectiveness were vaccine price, burden regarding pneumonia-related parameters, and the inclusion of herd effects. *Conclusion:* The children pneumococcal vaccination program appears to be a cost-effective intervention in Asia, and even cost-saving in certain conditions. Vaccine price, pneumonia-related disease burden, and the inclusion of the herd effect are observed as important key drivers in estimating cost-effectiveness in this region. Incorporating PCV in vaccination programs in this region was found to be highly favorable.

## 1. Introduction

Pneumococcal disease (PD), which is caused by *Streptococcus pneumoniae* (SP), remains to be a globally problematic burden, accounting for 16% of deaths in children less than five years of age [1,2]. SP is transmitted through the exposure to airborne droplets, when there is direct individual-to-individual contact. The carriage of SP is more prevalent in children compared to adults, with increasing colonization rates observed from birth until the age of 1–2 years [3,4]. Its manifestation includes invasive PD (IPD), such as meningitis, bacteremia, and sepsis, and non-invasive PD (NIPD), such as acute otitis media (AOM), pneumonia, bronchitis, and sinusitis [5,6,7].

Complexity in early diagnosis and the growing incidence of SP penicillin-resistant strains has complicated management of PD, contributing to a substantial clinical and economic burden on the health care system and in society [8,9,10]. Vaccination remains the cornerstone in reducing PD rates. Among 90 known serotypes of SP, various serotypes can lead to PD [2]. Protection against seven SP serotypes (4, 6B, 9V, 14, 18C, 19F, and 23F) is provided by the first pneumococcal conjugate vaccine (PCV) developed, a seven-valent PCV (PCV7). A significant reduction of PD incidence rates was associated with the use of this vaccine [11,12]. The licensure of newer versions of PCV, a 10-valent PCV (PCV-10) and a 13-valent PCV (PCV-13), which consist of an additional three (1, 5, 7F) and six (1, 3, 5, 6A, 7F, 19A) serotypes’ antigens, respectively, were assessed based on noninferiority efficacy comparison with PCV-7 [13,14]. With the proven effectiveness of PCV, the World Health Organization (WHO) encourages the prioritization of the inclusion of PCV in national universal immunization programs, worldwide [15].

The burden of PD is considerably high in the Asian region. Asian countries are among the countries with the highest number of deaths associated with PD. The incidences of PD is concentrated particularly in South Asia [2,16]. However, within Asian countries, only few countries, such as Pakistan and the Philippines, have already included PCV in their universal vaccination programs [17,18]. The exact policies in pneumococcal vaccination programs are usually determined on multiple factors, including (availability of) prevalence data, perception of vaccine effectiveness, and national budgets [18,19,20]. The role of a health-economic evaluation is prominent in the decision-making process related to public health interventions. It provides the evidence to ensure efficient allocation of scarce healthcare resources [19]. Such information may contribute to the evidence-based policy formulation related to PCV and help policy makers in the decision on the possible introduction of a universal PCV immunization program in the region. Considering the high burden of PD in Asia and the lack of a comprehensive review on a health-economic evaluation of PCV in this region, we aimed to summarize potential health and economic benefits of PCV for children in Asian countries.

## 2. Methods

The Preferred Reporting Items for Systematic Reviews and Meta Analyses (PRISMA) statement was used for this systematic review [21].

### 2.1. Search Strategy

The systematic search was conducted in two electronic databases (Pubmed and EMBASE) to identify all economic evaluation studies of PCV for children in Asian countries. The following keywords were used for the search: ("Pneumococcal Vaccines"[Mesh] OR PCV) OR (("Pneumonia"[Mesh]) AND (Vaccine* OR Immune*) AND (Costs and Cost Analysis OR Cost-Benefit Analysis OR Cost Effectiveness OR Cost Utility OR Cost Minimi* OR Economic Evaluation OR Economic Analysis) AND list of Asian Countries (for details see Supplementary Materials).

### 2.2. Study Selection

The initial search records from electronic databases were exported to Mendeley reference manager and checked for duplicates. The title and abstracts were then screened for relevance. The full texts of the included records were retrieved and reviewed. The screening process was performed by two reviewers (NZ and WNI) with the following inclusion criteria: Study design should be a complete economic evaluation classified in one of the formal health-economic study categories, including cost-minimization analysis (CMA), cost-effectiveness analysis (CEA), cost-utility analysis (CUA), or cost-benefit analysis (CBA); and the intervention should be a universal PCV immunization program for children in Asian countries. Any disagreements were resolved by discussions with a third reviewer (AAS).

We excluded multi-country economic evaluation studies without specific analysis per country, systematic review, experimental and observational studies, conference abstracts, and non-English studies.

### 2.3. Data Extraction

From each included study, we extracted data regarding study characteristics (author, year of publication, country, study objectives, detailed analysis, type of study), study design (methods, perspectives, herd effect, time horizon, discount rate, outcomes, and sensitivity analysis), and cost elements. Economic results from the analysis were converted to 2018 International $ using purchasing power parity (PPP) and gross domestic product (GDP) deflators [22,23]. If a study did not state the year of costing, it was estimated that the costing year was similar to the year of publication.

### 2.4. Quality Assessment

The quality of reporting was assessed using the Consolidated Health Economic Evaluation Reporting Standards (CHEERS) statement. It provides a set of recommendations, including a checklist, to facilitate adequate reporting of economic evaluation studies. The checklist consisted of 24 items classified in 6 categories, namely title and abstract, introduction, methods, results, discussion, and others. The compliance to each of the criteria was assessed for each study and categorized as follows: Complied completely, partially, or not at all [24].

## 3. Results

### 3.1. Systematic Search

**Figure 1:**
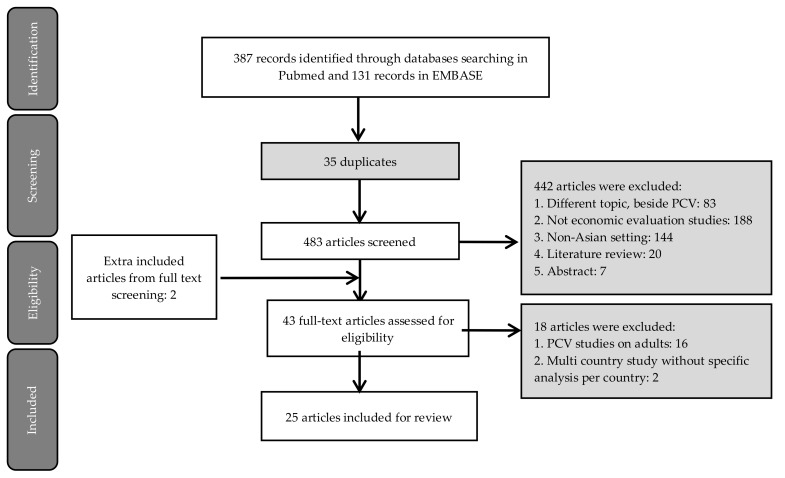
The Preferred Reporting Items for Systematic Reviews and Meta Analyses (PRISMA) flow diagram of study selection.

The initial search identified 518 records in Pubmed and Embase. After removing 35 duplicates, 483 articles were screened by title and abstract, excluding 442 records. Forty-three records screened on full text, of which 16 articles were excluded due to the study being conducted in adults (*n* = 16) and multi-country studies without a specific analysis per country (*n* = 2). Two extra additional articles were identified from snowballing on references during full-text screening, resulting in the final inclusion of 25 studies (Figure 1) [20,25,26,27,28,29,30,31,32,33,34,35,36,37,38,39,40,41,42,43,44,45,46,47,48].

### 3.2. Characteristics of Included Studies

The included studies were conducted in 11 Asian countries, including China (*n* = 7) [26,30,32,33,35,41,48] Malaysia (*n* = 3) [34,42,44], Hong Kong (*n* = 3) [28,29,42] Korea (*n* = 2) [25,46], The Philippines (*n* = 2) [31,36],Taiwan (*n* = 2) [27,43], Japan (*n* = 3) [20,38,39], Thailand (*n* = 1) [47], Mongolia (*n* = 1) [45], Bhutan (*n* = 1) [37], and India (*n* = 1) [40] (Table 1). One study was a multiple country analysis, performed in Malaysia and Hong Kong [42]. The oldest study that appeared from our search was from 2009, addressing the CEA of PCV-7 in Hong Kong [29], and the most recent one was from 2019, investigating the cost-effectiveness of PCV-13 in India [40]. At the time when the research was conducted, only three countries, i.e., Mongolia, Bhutan, and India [37,40,45], were eligible for discounted vaccine purchase prices offered through support from Gavi, The Vaccine Alliance, an organization established in 2000 to improve access to the vaccine for children in the world’s poorest countries [49].

#### 3.2.1. Study Design

The majority of the studies (*n* = 10) compared the potential benefits of two newer versions of PCV, namely PCV-10 and PCV-13 [20,25,28,31,34,36,37,42,44,47]. Seven studies assessed the cost-effectiveness of PCV-7 [27,29,30,32,33,39,46], of which one study assessed its effectiveness in the context of typical and pandemic influenza seasons [30]. PCV-13 was assessed in six studies [35,38,40,41,43,45] and comparisons of various types of vaccines were performed in two studies [26,48]. The majority of studies used quality-adjusted life years (QALYs) or disability-adjusted life years (DALYs) as the health outcomes measure [20,25,26,32,35,36,37,38,39,40,41,44,47,48] while the rest combined both the clinical and the utility outcomes or provided only clinical outcomes, e.g., cases of pneumococcal-related diseases and life years. Three studies performed both a CUA and a budget impact analysis (BIA) [31,37,45], while one study performed CEA and BIA [39].

All included studies used a decision analytic model to assess the health-economics benefits of PCV in Asia. In total, 18 studies used static models, of which 10 studies applied a Markov model [20,25,26,31,34,36,42,44,47,48] and 10 studies performed analysis in a decision-tree analytical model [27,28,30,32,33,40,41,45,46,50]. Only one study used a dynamic model with age-structured transmission [43]. The majority of the studies (*n* = 21) took the herd effect of vaccination into account.

Regarding the perspectives, seven studies adopted the societal perspective [26,32,38,39,46,47,48], four studies adopted both societal and payer perspectives [28,42,43,47], and two studies adopted both societal and health care perspectives [20,45]. The payer perspective alone was used in four studies [28,30,33,41] while the government perspective was adopted in six studies [25,34,36,37,40,44]. Details on the cost element, including the type of costs that were taken into account, are provided in Table 2.

The majority of the studies reported the discount rate for both costs and effects. Within the included studies, a 3% discount rate was adopted in the majority of the studies (*n* = 16) [20,26,27,32,33,37,38,39,40,41,42,43,44,47,48]. One study used a 3.5% discount rate [31], while another seven studies used a 5% discount rate [25,28,29,34,35,36,46]. One study reported the discount rate only for the cost, not for the effects (5%) [30]. None of the included studies used differential discounting for costs and effects. The majority of the studies used a long time horizon (10, 30, 75, and 100 years, and lifetime [25,26,27,28,29,31,34,35,36,42,43,44,45,47,48] and eight studies used a short time horizon (1 and 5 years) [20,30,32,33,37,38,39,41,51]. In the BIAs performed in four studies, 5- and 10-year time horizons were used [31,37,39,45].

#### 3.2.2. Cost Component

In the majority of the studies, direct medical costs included the treatment of pneumonia, meningitis, bacteremia, AOM (with and without myringotomy), as well as treatment for their sequelae, e.g., hearing loss (cochlear implant) and neurologic impairment [20,25,26,27,28,29,30,31,32,33,34,36,42,43,44,45,46,47,48]. Direct non-medical costs included transportation for PD therapy, meals, and accommodation, while indirect medical costs included the costs associated with economic productivity loss due to PD morbidity and/or mortality in patients or caregivers [20,27,46] (Table 2). The vaccine price per dose was reported by all included studies, with 15 studies additionally reporting the vaccine administration cost [20,25,26,28,29,30,31,32,33,34,36,37,40,42,43,44,45,46,47,48]. Within the included studies, the prices of PCV-7 were in the range of I$ 92.09–253.02. The highest price of PCV-7 was observed in two studies conducted in China [30,33]. The price of PCV-10 ranged from I$ 26.95, in a study performed in Hong Kong [42], to I$ 173.04, in a study performed in Malaysia [34]. The price of PCV-10 in Bhutan was I$ 3.71 while that of PCV-13 in Bhutan and Mongolia were I$ 3.56 and I$ 3.71, respectively, due to their eligibility to purchase vaccine through Gavi support [45]. One study conducted in India used a wide range of PCV-13 prices over a 10-year period (I$ 3.45–I$ 71.14), taking into account the transition after partnership with GAVI and subsidization by a pharmaceutical company. Among countries that were non-eligible to receive the Gavi subsidy, the price of PCV-13 was in the range of I$ 36.92–I$ 173.04. The vaccine administration costs varied between I$ 0.03 and I$ 37.74 (Table 3).

#### 3.2.3. Study Findings

The majority of the studies (*n* = 22) confirmed that pneumococcal vaccination would be a cost-effective intervention [20,25,26,27,28,29,30,31,33,34,35,36,37,38,39,40,41,42,43,44,45,48]. Only three studies showed that PCV-7, PCV-10, and PCV-13 would not be cost-effective [32,46,47]. There were conflicting results with regards to the benefits of PCV-10 and PCV-13. Five studies showed that PCV-10 would be cost-saving compared to PCV-13 [25,34,36,42,44], while another five studies found the opposite result [20,26,31,37,42]. Four studies confirmed that PCV-7 would be cost-effective compared with no vaccination, of which one study also showed that it would be cost-saving [27,29,30]. PCV-13 was assessed in six studies, with cost-effective findings observed in all studies [35,38,40,41,43,45], with an additional condition observed in a Japanese study showing that PCV-13 would be a socially acceptable option compared to current PCV-7 vaccination if it had additional protection against AOM compared to PCV-7, and the cost of PCV-13 per dose is 1.7 times less than that of PCV-7. Four BIAs showed conflicting results, in which two studies showed the introduction of PCV-13 would reduce healthcare costs as well as societal costs [37,45], while the other two showed that the cost of a universal PCV immunization program was higher than the current healthcare budget, which was only sufficient to provide PCV vaccination for 25% of the cohort [31] and either the no co-payment or co-payment vaccination program appears to not be budget saving for the first six years [39].

Findings on a favorable cost-effectiveness are related with the threshold of willingness to pay (WTP) to define cost-effectiveness. The majority of the studies (*n* = 17) used the WHO criteria [26,27,29,30,32,34,35,36,37,38,39,40,41,42,43,44,48], namely that vaccination would be considered cost-effective if the incremental cost-effectiveness ratio (ICER) was not more than three times the gross domestic product (GDP) per capita and cost-saving if it was not more than one time GDP per capita [52]. Five other studies used more conservative approaches, in which cost-effectiveness was defined as the ICER not exceeding one time GDP per capita [25,28,31,45,47]. One study explicitly stated the WTP threshold based on a national pharmacoeconomic guideline [20], while the other one synthesized a range of WTP thresholds based on an acceptability curve of vaccination with various vaccine prices per dose and dosing schedules [46].

All included studies performed one-way sensitivity analysis, of which 14 studies performed additional probabilistic sensitivity analysis (PSA) [20,25,31,32,35,37,38,42,43,44,46,47,48], and two studies conducted two-way sensitivity analysis and PSA [26,28]. The most sensitive parameters observed included vaccine price [27,29,31,32,43]; AOM-related parameters, e.g., disutility of AOM patients, cost for inpatient myringotomy, AOM GP visits, and vaccine efficacy against AOM [20,25,26,28,34,38,39]; pneumonia-related parameters, e.g., incidence of pneumonia, cost for inpatient pneumonia, and vaccine efficacy against pneumonia [35,36,41,42,43,48,51]; the and inclusion of the herd effect [27,30,31,33,50] (Table 2).

#### 3.2.4. Quality of Reporting

**Figure 2:**
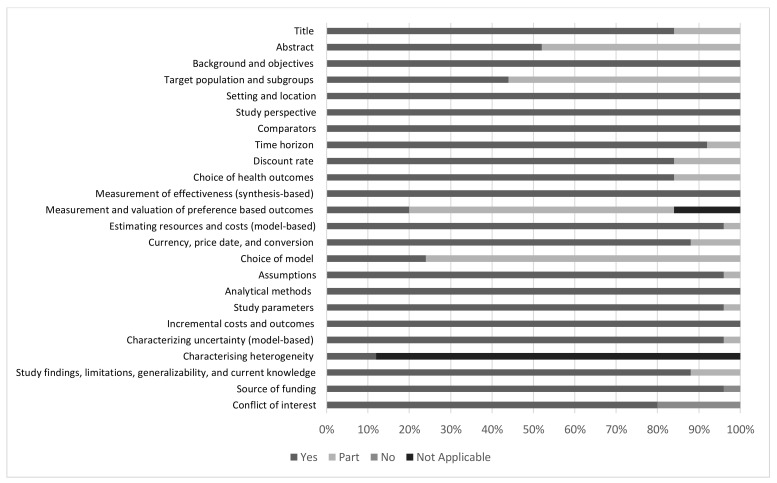
The result of the Consolidated Health Economic Evaluation Reporting Standards (CHEERS) assessment.

Fulfilment of the reporting criteria based on the CHEERS checklist varied among the sections. Sections that were sufficiently reported by all studies included the introduction, study perspective, setting, and comparator. In the abstract section, almost one-third of the articles failed to report brief results of the sensitivity analysis. Most articles described the target population and time horizon for vaccination, but the reason for choosing such a group and time range were partly reported. The discount rate for both vaccine price and efficacy were sufficiently reported by most studies. CHEERS recommends the reporting of the outcomes and the reason for choosing the measure. The latter criterion was not sufficiently reported. Most studies reported the source of costs estimation, such as from the authority data and published studies. The currency used was provided in all studies, but the year of costing and conversion were not fully reported. Measurement and valuation of preference-based outcomes, such as health-related quality of life (HRQoL), were another criterion that was poorly reported by relevant articles. Furthermore, we found that although most studies reported the choice of economic models, only a few reported the rationale to use such a model. In the discussion section, most studies reported information related to the generalizability and study limitations. The source of funding was reported in most studies, but not all studies described the role of the funder. A summary of the results from the CHEERS checklist is provided in Figure 2.

## 4. Discussion

This systematic review demonstrated that a pneumococcal vaccination program for children would be a cost-effective intervention in most Asian countries (in 22 of 25 studies), and even cost-saving in certain conditions. Potentially comparable benefits were shown by PCV-10 and PCV-13, with five studies showing that PCV-10 would be cost-saving compared to PCV-13 [25,34,36,42,44], and another five studies favoring PCV-13 [20,26,31,37,42]. The findings were sensitive to vaccine price, AOM, and other diseases’ burden regarding pneumonia-related parameters, and the inclusion of the herd effect.

Most of the decision analytic models used in this review were static models. Herd effects could have a significant impact on health economic analysis of PCV. In this review, most studies considered herd effects, although the majority of studies incorporated this effect in a static model, and only one study assessed it in a dynamic model. The application of a dynamic model allows better estimation of disease exposure that is related to the development of the herd effect. In countries where the vaccine coverage is particularly high, a static model may be sufficient due to a lack of further herd immunity benefits since most individuals are already vaccinated [53,54,55]. However, as most Asian countries have considerably low PVC coverage [17], incorporating a dynamic model, where disease transmission is comprehensively taken into account by indirect effects, such as herd protection, can better capture the benefit of PCV in a population. Real-world herd effects following the introduction of PCV-13 on IPD were observed in different regions, such as in the USA, Denmark, France, and the UK, where high coverage of the vaccine was observed [56].

Costs related to PCV, as one of the driving factors of cost-effectiveness, varied across the countries in the included studies. Multiple factors can influence vaccine prices, including regulation for procuring vaccines, public health value, and the extent of the government’s commitment related to coverage of the vaccine for the population [57]. In several Asian countries, PCV is only available on the private market, such as in Malaysia and China, resulting in a higher price of PCV [28,42]. In a Chinese study showing that PCV-7 would not be cost-effective, the price of PCV was even more expensive than in European vaccination programs [32,58]. Although in most studies the PCV was proven to be cost-effective, we observed in several countries that the additional budget to healthcare should be allocated to allow the inclusion of PCV in their universal immunization program [26,31]. It was estimated that PCV would cost more than any other vaccines in such a program [38]. Negotiations with manufacturers should be initiated to obtain better pricing, enabling better immunization coverage and greater market sustainability of PCV.

One of the major contributing factors in PD burden are related to AOM. Previous studies indicated that the burden and expenses for AOM treatment could exceed that of IPD [59]. Although AOM is not life-threatening, it is highly prevalent and its treatment, including the sequelae, requires a substantial amount of costs due to the high volume of patient consultation, myringotomy/tympanostomy tube surgical procedure, and utilization of antibiotics [59,60]. Vaccine protection against AOM and pneumonia was also frequently reported as an influential parameter on PCV cost-effectiveness [20,25,28,44].

A previous systematic review on the cost-effectiveness of pneumococcal vaccination in children in low- and middle-income countries (LMICs) suggested that PCV vaccination in children was considered to be a cost-effective intervention in most LMICs. Similar to our results, the key drivers of cost-effectiveness results were vaccine price, burden concerning pneumonia-related parameters, and vaccine efficacy [61]. Among 22 included studies in the aforementioned review, only 7 Asian countries were included. Considering that the burden of PD is considered high in the Asia region, our current review can complement the results from a previous systematic review to inform decisions makers on the costs and benefits of introducing PCV vaccination in a country’s immunization program.

A global modelling analysis assessing both the effect and cost-effectiveness of PCV vaccination predicted that the introduction of PCV vaccination was estimated to be the most effective in averting disability-adjusted life years (DALYs) in Asia and Africa [62], which is probably due to the high burden of pneumonia-related diseases in both regions. The ICER for PCV introduction was also estimated to be cost-effective in the majority of countries worldwide [62], as indicated by cost-effectiveness thresholds, such as GDP per capita, and a more stringent one using the country-level opportunity cost of health expenditure [63]. Nevertheless, PCV is one of the most expensive vaccines, which can hamper its introduction, especially in countries with limited resources. Vaccine price remains one of the important key drivers of cost-effectiveness in many countries [61,63]; therefore, reviving joint efforts, especially on PCV introduction with affordable prices, is necessary, especially for underprivileged populations [62]. This review indicates that a comparable cost-effectiveness is observed among PCV-10 and PCV-13 in several different settings, suggesting that a country’s decision to incorporate PCV into its immunization program should also evaluate the interchangeability of the vaccine by considering benefits in terms of both the cost and effectiveness. This could enable policymakers to make an informed decision while choosing the most appropriate vaccine according to the country’s epidemiological and immunization program [64].

In this review, the CHEERS checklist was used to assess the quality of reporting in the included studies. The quality of reporting is one of the prominent aspects of economic evaluation studies, as it provides transparency. Although most of the studies were already adequately complied with the standards in the checklist, there were some points there were poorly fulfilled by the majority of studies, e.g., the rationale to use the chosen decision modeling and the details or description of the role of the funder. Previous studies observed that health economic evaluation funded by pharmaceutical companies tends to have favorable results compared to noncommercially funded studies, thus the reporting of the source of funding and its role in the study is important to allow better assessment of study credibility [65,66].

To the best of our knowledge, this is the first systematically performed review on PCV with the focus on Asia. Systematic approaches were taken in identifying relevant studies from electronic databases. Furthermore, the initial and full-text screening process were carried out by two researchers independently using prespecified inclusion and exclusion criteria, therefore reducing potential bias.

Inevitably, this review has some potential limitations. Although systematic approaches were applied in both the literature search and screening, there is a possibility that some studies may were missed as we focused more on studies in peer-reviewed journals, published in the English language. CHEERS checklist was used to assess the reporting format of the included studies, and the compliance to each criteria in the checklist was categorized as complete, partial, or not at all. However, in the end, the classification and also interpretation were based solely on the reviewers, who did do their utmost best to be highly objective in their task.

## 5. Conclusions

As pneumococcal infections result in a considerable burden in the Asian region, the control of PD with vaccination is of utmost importance. Some prominent parameters, such as the vaccine price, pneumonia-related burden of disease, and the inclusion of the herd effect in the analysis, were observed as key drivers for estimating the cost-effectiveness in this region. A pneumococcal vaccination program for children appeared to be a cost-effective intervention in the Asian region, and even cost-saving under certain conditions.

## Figures and Tables

**Table 1:** General characteristics of economic evaluations on Pneumococcal Conjugate Vaccine (PCV) in Asian countries.

Author, Year	Country	Model Type	Type of Vaccine(s)	Time Horizon	Inclusion of Herd Effect	Outcome Measure(s)	Sensitivity Analysis
Lee et al., (2009) [29]	Hong Kong	Decision tree	PCV-7	10 year	Yes	LYs	One-way
Sohn et al., (2010) [46]	Korea	Decision tree	PCV-7	5 year	No	LYs	One-way and PSA
Wu et al., (2012) [43]	Taiwan	Age-structured transmission dynamic model	PCV-13	10 year	Yes	LYs	One-way and PSA
Hoshi et al., (2012) [39]	Japan	Markov	PCV-7	5 year	Yes	LYs and QALYs	One-way
Lee et al., (2013) [28]	Hong Kong	Decision tree	PCV-10 and PCV-13	10 year	Yes	LYs and QALYs	One-way, two-way, and PSA
Kulpeng et al., (2013) [47]	Thailand	Markov	PCV-10 and PCV-13	Lifetime	Yes	QALYs	One-way and PSA
Wu et al., (2013) [27]	Taiwan	Decision tree	PCV-7	10 year	Yes	LYs	One-way
Hoshi et al., (2013) [38]	Japan	Markov	PCV-13	5 year	Yes	LYs and QALYs	One-way and PSA
Shiragami et al., (2014) [20]	Japan	Markov	PCV-10 and PCV-13	5 year	Yes	QALYs	One-way and PSA
Zhang et al., (2014) [36]	The Philippines	Markov	PCV-10 and PCV-13	Lifetime	Yes	QALYs	One-way and PSA
Hu et al., (2014) [30]	China	Decision tree	PCV-7	1 year	Yes	LYs, QALYs, pneumonia-related Illness	One-way
Aljunid et al., (2014) [34]	Malaysia	Markov	PCV-10 and PCV-13	Lifetime	Yes	LYs and QALYs	One way
Che et al., (2014) [32]	China	Decision-tree	PCV-7	5 year	Yes	QALYs	One-way and PSA
Haasis et al., (2015) [31]	The Phillipines	Markov	PCV-10 and PCV-14	Lifetime (CUA) and 5 year (BIA)	Yes	QALYs	One way
Caldwell et al., (2015) [33]	China	Decision tree	PCV-7	1 year	Yes	LYs and QALYs	One-way
Maurer et al., (2016) [26]	China	Markov	PCV-7, PCV-10, and PCV-13	Lifetime	Yes	QALYs	One way, two-way, and PSA
Mo et al., (2016) [48]	China	Markov	PCV 7, PCV 13, PPV 23	Lifetime	Yes	QALYs, mortality	One-way and PSA
Wu et al., (2016) [42]	Malaysia and Hong Kong	Markov	PCV-10 and PCV-13	10 year	Yes	LYs and QALYs	One-way and PSA
Sundaram et al., (2017) [45]	Mongolia	Age-stratified decision tree	PCV-13	30 year (CEA) 10 year (BIA)	Yes	DALYs	One-way
Wang et al., (2017) [44]	Malaysia	Markov	PCV-10 and PCV-13	10 year	No	QALYs	One-way and PSA
Zhang et al., (2018) [25]	Korea	Markov	PCV-10 and PCV-13	10 year	No	QALYs	One-way and PSA
Dorji et al., (2018) [37]	Bhutan	Markov	PCV-10 and PCV-13	1 year	Yes	QALYs, pneumococcal-related Illness, mortality	One-way and PSA
Zhou et al., (2018) [35]	China	Markov	PCV-13	Lifetime	Yes	QALYs	One-way and PSA
Shen et al., (2018) [41]	China	Decision tree	PCV-13	1 year	Yes	LYs, QALYs, mortality	One-way
Khrisnamoorty et al., (2019) [40]	India	Decision tree	PCV-13	10 year	No	DALYs, pneumococcal-related Illness, mortality	PSA

CEA: Cost-Effectiveness Analysis, CUA: Cost-Utility Analysis, BIA: Budget Impact Analysis, PCV: Pneumococcal Conjugate Vaccine, PPV: Pneumococcal Polysaccharide Vaccine, LYs: Life Years, QALYs: Quality-Adjusted Life Years, DALYs: Disability-Adjusted Life Years, PSA: Probabilistic Sensitivity Analysis.

**Table 2:** Cost elements and main findings of economic evaluations on PCV vaccination in Asian countries.

Reference	Perspective	Discount Rates		Costs Data			Willingness to Pay Threshold (International $ 2018)	Main Findings	Most Influential Parameter in Sensitivity Analysis
		Cost	Outcome	Direct Costs		Indirect Costs			
				Medical	Non-Medical				
[29]	Payer and Societal	5%	5%	Direct acute cost per infection, and long-term cost per disability	Transportation cost for outpatient visits and hospital stays	Productivity loss of caregiver	1–3 times GDP per capita	PCV-7 would be cost saving compared to no vaccination	Vaccine cost, herd effect, and incidence of pneumonia
[46]	Societal	5%	5%	Treatment of pneumococcal diseases and clinical sequelae (e.g., prescribed medications, medical procedures, and diagnostic tests)	Transportation cost for outpatient visits and hospital stays	Productivity loss of caregiver owing to pneumococcal disease morbidity	ICER below 360,000	PCV-7 would not be cost-effective compared to no vaccination	Annual incidence of pneumonia, AOM, and bacteremia
[43]	Payer and Societal	3%	3%	Hospitalization, health-care professional consultation, ICU admissions, medications, and diagnostic tests for IPD, pneumonia, AOM	NR	Productivity loss of patient due to non-fatal pneumococcal diseases and pneumococcal death and caregiver time	1–3 times GDP per capita	PCV-13 would be cost-effective	Vaccine price, recovery rate, incidence of pneumonia and the vaccine-immunity period
[39]	Societal	3%	3%	Treatment of pneumococcal diseases and meningitis sequelae	NR	Productivity loss of caregiver for vaccine uptake and medical treatment, and for taking care of a child with sequelae	1–3 times GDP per capita	PCV-7 would be cost-effective	Vaccine effectiveness in reducing AOM, care-giver’s productivity loss
[28]	Payer	5%	5%	Pneumococcal-related disease cost, hospitalization, outpatient cost of pneumonia, and AOM (e.g., GP and complications)	NA	NA	1 time GDP per capita	PCV-10 would be cost-saving compared to PCV-13	Cost for inpatient myringotomy and changes in AOM-Related parameters
[47]	Societal	3%	3%	Treatment cost per episode of meningitis, hospitalization due to pneumonia-related illness	Transportation and accommodation	Productivity loss of caregiver	1 time GDP per capita	PCV10 and PCV13 would not be cost-effective	Discount rate, change in duration of vaccine protection, and the incidence of pneumonia
[27]	Payer and Societal	3%	3%	Treatment of pneumococcal-related disease (i.e., hospitalization, healthcare professional consultation fees, intensive care admissions, medications, and diagnostic tests)	NR	Productivity loss of patient due to non-fatal pneumococcal diseases and pneumococcal death and caregiver time	1–3 times GDP per capita	PCV-7 would be cost effective compared to no vaccination	Vaccine price, herd effect on pneumonia, and mortality rate of pneumonia
[38]	Societal	3%	3%	Treatment of pneumococcal diseases and meningitis sequelae	NR	Productivity loss of caregiver for vaccine uptake and medical treatment, and for taking care of a child with sequelae	1–3 times GDP per capita JPY	PCV-13 would be a socially acceptable option compared to current PCV-7 vaccination if PCV-13 had additional protection against AOM compared to PCV-7 and cost PCV-13 per dose is 1.7 times less than that of PCV-7	Vaccine effectiveness in reducing AOM
[20]	Health care provider and societal	3%	3%	Treatment cost, admission days and outpatient visits	NR	Wages lost due to acute episodes	ICER below 45.000	PCV-10 would be cost saving compared to PCV-13	PCV-10 efficacy against AOM, percentage reduction in myringotomy, and changes in other AOM-related parameters
[36]	Government	5%	5%	Hospitalization, inpatient/outpatient diagnostic tests, medication/vaccine costs, and health care professionals’ fees	NA	NA	1–3 times GDP per capita	PCV-10 would be cost effective compared with no vaccination and cost-saving compared to PCV-13	Percent reduction in CAP hospitalization, hospitalization for CAP, and vaccine efficacy
[30]	Payer	5%	5%	Hospitalization and physician consultation fees, diagnostic tests, and medication expenses for pneumococcal-related diseases	NA	NA	Max 3 times GDP per capita	PCV-7 would be cost-effective	Percent reduction in disease incidence among the unvaccinated population, vaccine price, vaccine coverage level
[34]	Government	5%	5%	Outpatient treatment and hospitalization cost	NA	NA	1 time GDP per capita	PCV-10 would be cost effective compared to no vaccination and cost-saving compared to PCV-13	GP visits for AOM and PCV-10 efficacy
[32]	Societal	3%	3%	Medical cost of meningitis, bacteremia, pneumonia, AOM, long-term cost of sequelae	NR	Productivity loss of caregiver	1 time GDP per capita	PCV-7 would not be cost effective	Cost of PCV-7 per dose, the reduction of IPD for herd immunity in adults and annual incidence of IPD in children
[31]	Health system	3,5%	3,5%	Cost per episode of meningitis, bacteremia and sepsis, all-cause pneumonia hospitalization, all-cause pneumonia outpatients	NA	NA	1 time GDP per capita	CUA: both PCV-10 and PCV-13 would be cost-effective compared to vaccination. PCV13 achieved better value for money compared to PCV10. BIA: cost of national PCV immunization program is expected to be higher than current healthcare budget.	Vaccine cost, exclusion of herd effect, and vaccine efficacy
[33]	Payer	3%	3%	Hospitalization, physician consultation, diagnostic tests, nursing and medication expenses for all-cause pneumonia and pneumococcal-related illness	NA	NA	1–3 times GDP per capita	PCV7 would be cost-effective during a typical influenza season and cost-saving during an influenza pandemic	Variation in the herd effect and vaccine coverage
[26]	Payer	3%	3%	Treatment of pneumonia-related illness and complications	_	NR	1–3 times GDP per capita	PCV-13 would be cost-saving compared to PCV-7 and PCV-10	Utility of AOM, the cost of PCV-13, incidence of pneumonia and AOM
[48]	Societal	3%	3%	Treatment of pneumonia-related illness	NR	NR	1–3 times GDP per capita	PPV-23 would be the most cost-effective vaccine, followed by PCV-13	Efficacy of PPV-23 against pneumonia, cost of PCV-13, and cost of PCV-7
[42]	Payer and Societal	3%	3%	Treatment of pneumococcal-related illness and lifetime cost of meningitis sequelae	NR	Productivity loss	1–3 times GDP per capita	PCV-13 would be cost saving compared to PCV-10, under both payer and societal perspective in both countries	In Malaysia: PCV-10 and PCV-13 coverage In Hongkong: direct cost of treating hospitalized pneumonia and case-fatality ratio (CFR) of hospitalized pneumonia In Malaysia: PCV-10 and PCV-13 coverage In Hongkong: direct cost of treating hospitalized pneumonia and CFR of hospitalized pneumonia
[45]	Health system and societal	3%	3%	Hospitalization and health center consultation costs	NR	Productivity loss	1 time GDP per capita	CEA: PCV-13 would be cost-effective compared to no vaccination. BIA: PCV-13 would reduce direct cost to the healthcare budget and societal cost	Vaccine serotype coverage, disease burden, vaccine efficacy
[44]	Government	3%	3%	Hospitalization due to pneumonia-related illness and complications and GP consultation	NA	NA	1–3 times GDP per capita	PCV-10 would be cost effective compared to no vaccination and cost-saving compared to PCV-13	PCV-10 efficacy against AOM and disutility weight for AOM
[25]	Government	5%	5%	Hospitalization and outpatient due to pneumonia-related illness and GP consultation	NA	NA	1 time GDP per capita	PCV-10 would be cost-saving compared to PCV-13	Disutility for outpatient AOM, PCV-13% reduction in myringotomy, and GP visits for AOM.
[37]	Government	3%	3%	Treatment of pneumococcal-related illness, hospitalization due to pneumonia, and treatment of meningitis sequelae	NA	NA	1 time GDP per capita	Both PCV-10 and PCV-13 are cost-effective, with PCV-13 yields better health outcomes in terms of episodes of pneumococcal disease, number of deaths, and would incur a lower five-year budget.	Variation in coverage, duration of vaccine protection, excluding indirect vaccine effects (herd protection), and discount rate
[35]	Health system and societal	5%	5%	Treatment of pneumococcal-related illness and lifetime cost of meningitis sequelae	NR	Productivity lost	1 time GDP per capita	PCV-13 would be cost effective	CAP-related parameters (annual incidence of CAP, case-fatality of hospitalized CAP and S. pneumoniae isolation rate for pneumonia) and cost of PCV-13
[41]	Payer	3%	3%	Treatment of pneumococcal-related illness, all-cause otitis media, and hospitalization due to pneumonia	NA	NA	1–3 times GDP per capita	PCV-13 would be cost-effective at a threshold of 1–3 GDP per capita when considering direct vaccine effects only or indirect effects for rare invasive disease cases only. When indirect effects for the more frequently occurring inpatient pneumonia was included, the results are highly cost-effective at 1 times GDP per capita	incidence rates of inpatient pneumonia
[40]	Government	3%	3%	Hospitalization and outpatient due to pneumococcal-related illness	NA	NA	1 time GDP per capita	PCV-13 would be cost effective	Vaccine cost

NA: Not Available, NR: Not Reported, GDP: Gross Domestic Product, ICER: Incremental Cost-Effectiveness Ratio, CAP: Community-Acquired Pneumonia, GP: General Practitioner, PCV: Pneumococcal Conjugate Vaccine, AOM: Acute Otitis Media, ICU: Intensive Care Unit, IPD: Invasive Pneumococcal Disease, CFR: Case Fatality Rate.

**Table 3:** Vaccine price per dose and administrative cost of PCV in Asian countries (international $ 2018).

Author, Year/Country	PCV7	PCV10	PCV13	PPV23	Administration Cost
Lee, 2009/Hongkong	112.52	−	−	−	2.72
Sohn, 2010/Korea	94.18	−	−	−	−
Wu, 2012/Taiwan	−	−	107.01	−	5.49
Hoshi, 2012/Japan	92.09	−	−	−	−
Lee, 2013/Hong Kong	−	52.64	52.64	−	−
Kulpeng, 2013/Thailand	−	127.56	170.97	−	(6.38–8.55)
Wu, 2013/Taiwan	−	91.55	−	−	5.48
Hoshi, 2013/Japan	92.34	−	120.04	−	−
Shiragami, 2014/Japan	−	69.67	69.67	−	37.74
Zhang, 2014/The Philippines	−	48.51	48.51	−	−
Hu, 2014/China	253.02	−	−	−	2.94
Aljunid, 2014/Malaysia	−	173.04	173.04	−	−
Che, 2014/China	146.25	−	−	−	2.14
Haasis, 2015/The Philippines	−	36.73	41.20	−	−
Caldwell, 2015/China	253.02	−	−	−	2.94
Maurer, 2016/China	157.37	157.37	157.37	−	2.16
Mo, 2016/China	147.14	147.14	−	32.51	1.71
Wu, 2016/Malaysia-Hongkong	−	59.29 (Malaysia) 26.95 (Hongkong)	59.29 (Malaysia) 44.19 (Hongkong)	−	−
Sundaram, 2017/Mongolia	−	−	3.56	−	0.15
Wang, 2017/Malaysia	−	36.92	36.92	−	−
Zhang, 2018/Korea	−	57.16	57.16	−	18.53
Dorji, 2018/Bhutan	−	3.19	3.71	−	3.91
Zhou, 2018/China	−	−	~61.45	−	−
Shen, 2018/China	−	−	172.44	−	−
Krishnamoorthy, 2019/India	−	−	3.45–71.14	−	0.03

PCV: Pneumococcal Conjugate Vaccine, PPV: Pneumococcal Polysaccharide Vaccine.

## Supplementary Materials

The following are available online at https://www.mdpi.com/2076-393X/8/3/426/s1. Supplementary Material.
