# Supplementary material for: Pneumococcal Vaccination for Children in Asian Countries: A Systematic Review of Economic Evaluation Studies
Source: Vaccines (Basel). 2020 Jul 30;8(3):426. doi: 10.3390/vaccines8030426 (PMC7564215; doi:10.3390/vaccines8030426)
Supplement: Supplementary file 1 [file vaccines-08-00426-s001.zip › Supplementary Material Search Strategy.pdf]

Supplementary Material
Appendix I. Search Strategy

Search strategy

Vaccine term:

("Pneumococcal Vaccines"[Mesh] OR PCV) OR (("Pneumonia"[Mesh]) AND (Vaccine* OR Immune*)

Cost term:

AND ("Costs and Cost Analysis"[Mesh] OR "Cost-Benefit Analysis"[Mesh] OR Cost Effectiveness OR Cost Utility OR Cost Minimi* OR Economic Evaluation OR Economic Analysis)

Countries: (Asia)

AND ("Asia"[Mesh] OR "Asia, Central"[Mesh] OR "Kazakhstan"[Mesh] OR "Kyrgyzstan"[Mesh] OR "Tajikistan"[Mesh] OR "Turkmenistan"[Mesh] OR "Uzbekistan"[Mesh] OR "Asia, Southeastern"[Mesh] OR "Brunei"[Mesh] OR "Cambodia"[Mesh] OR "Indonesia"[Mesh] OR "Laos"[Mesh] OR "Malaysia"[Mesh] OR "Myanmar"[Mesh] OR "Philippines"[Mesh] OR "Singapore"[Mesh] OR "Thailand"[Mesh] OR "Timor-Leste"[Mesh] OR "Vietnam"[Mesh] OR "Asia, Western"[Mesh] OR "Bangladesh"[Mesh] OR "Bhutan"[Mesh] OR "India"[Mesh] OR "Nepal"[Mesh] OR "Pakistan"[Mesh] OR "Sri Lanka"[Mesh] OR "Middle East"[Mesh] OR "Afghanistan"[Mesh] OR "Bahrain"[Mesh] OR "Iran"[Mesh] OR "Iraq"[Mesh] OR "Israel"[Mesh] OR "Jordan"[Mesh] OR "Kuwait"[Mesh] OR "Lebanon"[Mesh] OR "Oman"[Mesh] OR "Qatar"[Mesh] OR "Saudi Arabia"[Mesh] OR "Syria"[Mesh] OR "Turkey"[Mesh] OR "United Arab Emirates"[Mesh] OR "Yemen"[Mesh] OR "Far East"[Mesh] OR "China"[Mesh] OR "Japan"[Mesh] OR "Korea"[Mesh] OR "Democratic People's Republic of Korea"[Mesh] OR "Republic of Korea"[Mesh] OR "Taiwan"[Mesh] OR "Asia, Northern"[Mesh] OR "Russia"[Mesh])

PUBMED

((Pneumococcal Vaccines* OR PCV) AND Pneumonia AND (Vaccine* OR Immune*) AND (Costs and Cost Analysis OR Cost-Benefit Analysis OR Cost Effectiveness OR Cost Utility OR Cost Minimi* OR Economic Evaluation OR Economic Analysis))

EMBASE

(pneumococcal AND vaccines* OR pcv) AND ('pneumonia'/exp OR pneumonia) AND (vaccine* OR immune*) AND ((((((costs AND ('cost'/exp OR cost) AND ('analysis'/exp OR

analysis) OR 'cost benefit'/exp OR 'cost benefit') AND ('analysis'/exp OR analysis) OR 'cost'/exp OR cost) AND effectiveness OR 'cost'/exp OR cost) AND utility OR 'cost'/exp OR cost) AND minimi* OR economic) AND ('evaluation'/exp OR evaluation) OR economic) AND ('analysis'/exp OR analysis)
